# Different patterns of myocardial iron overload by multislice T2* Cardiovascular MR as markers of risk for cardiac dysfunction in thalassemia major

**DOI:** 10.1186/1532-429X-13-S1-P309

**Published:** 2011-02-02

**Authors:** Antonella Meloni, Pasquale Pepe, Maria Chiara Dell'Amico, Gennaro Restaino, Valeri Gianluca, Massimo Midiri, Petra Keilberg, Angela Ciancio, Maria Lucia Boffa, Vincenzo Positano, Massimo Lombardo, Alessia Pepe

**Affiliations:** 1 “G. Monasterio” Foundation and Institute of Clinical Physiology, CNR, Pisa, Italy; 2 Università Cattolica del Sacro Cuore, Campobasso, Italy; 3 Azienda Ospedaliero-Universitaria Ospedali Riuniti, Ancona, Italy; 4 Policlinico "Paolo Giaccone", Palermo, Italy; 5 Ospedale “Madonna delle Grazie”, Matera, Italy; 6 Ospedale "S. Giovanni Bosco", Napoli, Italy

## Background

The multislice multiecho T2* CMR technique allows to detect different patterns of myocardial iron overload (MIO). The aim of this study was to verify the risk of biventricular dysfunction related to different patterns of MIO in a large cohort of thalassemia major (TM) patients.

## Methods

1135 TM patients (538 M, mean age 30 ± 19 years) enrolled in the Myocardial Iron Overload in Thalassemia (MIOT) network underwent CMR (1.5 T). For the assessment of MIO, three short-axis views of the left ventricle (LV) were acquired and the myocardium was segmented into 16-segments standardized LV model. The T2* value on each segment was calculated as well as the global T2* value. A conservative cut off of 20 ms was considered the limit of normal for the segmental and global T2* values. Biventricular function parameters were quantitatively evaluated by SSFP cine images. The lower limit of normal for the LVEF and the RVEF were established from CMR in a large cohort of well-treated TM patients without myocardial iron overload and cardiac diseases.

## Results

Four groups of patients were identified: homogeneous MIO (all segments with T2* values <20 ms) (N=173, 15%), heterogeneous MIO (some segments with T2* values≥20 ms and other segments with T2* values < 20 ms) and global heart T2*<20 ms (N=160, 14%), heterogeneous MIO and global heart T2*≥20 ms (N=337, 30%) and no MIO (all segments with T2* values≥ 20 ms) (N=465, 41%). The LV ejection fraction (EF) was significant different among the groups (P<0.0001) (figure 1A). Odds Ratio for LV dysfunction (LV EF<57%) was 4.8 (3.1-7.3 OR 95% CI; P<0.0001) for patients with homogeneous MIO vs patients with no MIO and 1.9 (1.2-3.2 OR 95% CI; P=0.007) for patients with heterogeneous MIO and global heart T2*<20 vs patients with no MIO. The right ventricular (RV) EF was significant different among the groups (P<0.0001) (figure 1B). Odds Ratio for RV dysfunction (RV EF<55%) was 2.1 (1.4-3.2 OR 95% CI; P=0.001) for patients with homogeneous MIO vs patients with no MIO.

## Conclusions

Biventricular dysfunction is correlated with MIO distribution decreasing from the patients with homogeneous MIO to the patients with no MIO. Homogeneous MIO and heterogeneous MIO with a global heart T2*<20 predicts a significantly higher risk to develop cardiac dysfunction suggesting an intensive chelation therapy in this group of patients.

